# Radical resection and reconstruction of the sternum for metastasis of hepatocellular carcinoma

**DOI:** 10.1186/s13019-020-01247-3

**Published:** 2020-07-29

**Authors:** Young Chul Yoon, Jiyun Lee, Jin Yong Jeong

**Affiliations:** 1 Department of General Surgery, Incheon St. Mary’s Hospital, College of Medicine, The Catholic University of Korea, Seoul, Republic of Korea; 2 Department of Thoracic and Cardiovascular Surgery, Incheon St. Mary’s Hospital, College of Medicine, The Catholic University of Korea, 56 Dongsu-ro, Bupyeong-gu, Incheon, 21431 Republic of Korea

**Keywords:** Resection, Reconstruction, Sternum, Metastasis, Malignancy

## Abstract

Metastatic hepatocellular carcinoma of the sternum is rare and a few cases of surgical resection have been reported. Anterior chest wall reconstruction after radical resection of the sternum and ribs aims to protect the heart and lung from external damage and herniation and restore physiologic stability of the chest wall during respiration. A variety of reconstruction techniques using various materials have been reported, but so far there are no definitive guidelines for the reconstruction of chest wall defects. Recently, we encountered a rare case of metastatic cancer of the sternum from hepatocellular carcinoma in which radical resection of the sternum and ribs, and anterior chest wall reconstruction with acellular dermal matrix and titanium plates were performed.

**Dear Sir,**

With great interest, we read the report by Motono et al. presenting a case of sternal resection and reconstruction for metastasis from breast cancer [1]. Metastatic tumors of the sternum are uncommon. They usually originate from breast cancer, malignant melanoma, colorectal cancer, renal cell cancer, and cervical cancer [2]. Radical resection should be performed for curative intent by achieving a tumor-free margin for metastatic sternal tumors [2]. The authors performed surgical resection with curative-intent and filled the defect by sandwiching molded methylmethacrylate between polypropylene meshes. A majority of hepatocellular carcinoma (HCC) metastases occur in the lung, lymph nodes, bones, and adrenal glands [3]. Frequent bone metastasis sites of HCC are the spine, pelvic bone, ribs, long bones, and skull. Metastatic HCC of the sternum is rare [4]. Recently, we encountered a rare case of metastatic cancer of the sternum from HCC, for which radical resection of the sternum and ribs, and anterior chest wall reconstruction with acellular dermal matrix and titanium plates were performed.

An 80-year-old female presented with anterior chest pain for a month. She had undergone a laparoscopic posterior sectionectomy of the liver due to a 4-cm-sized hepatitis B-related HCC 1.5 years ago. Adjuvant systemic chemotherapy was not performed. Physical examination showed tenderness on the lower half of the sternum with normal external appearance. Tumor marker values were within normal ranges (serum alpha-fetoprotein, 2.92 ng/mL; prothrombin induced by vitamin K absence-II, 23.9 mAU/mL). Chest computed tomography scans revealed a 7.0 × 4.5 × 2.7 cm sized enhancing soft tissue mass with destruction of the sternum in the anterior chest wall (Fig. 1a). Whole-body bone scintigraphy demonstrated a photon defect accompanied by rim activity in the lower sternum (Fig. 1b). We decided to conduct a radical sternal resection with curative intent because the patient had symptoms with only a single metastasis., In the absence of definitive surgical resection, it has been shown that medical treatment can result in survival of less than 1 year [4]. We performed surgical resection in a supine position. The sternal tumor was clearly distinguished from the deep muscle fascia. Radical resection was performed. Margins of the frozen tissue section were confirmed to be negative. Radical resection was performed with lower two-thirds of the sternum, including the tumor, bilateral ribs (part of the third costal cartilage - 7th costal cartilage), and pericardial fat around the tumor. The defect of the anterior chest wall was covered with acellular dermal matrix (12 × 12 cm; MegaDerm®, L&C BIO, Seoul, Korea) over the pericardium (Fig. 1c). Additional skeletal reinforcement was performed with titanium plates (RibFix Blu™, MIMMER BIOMET, Jacksonville, FL, USA) to stabilize the chest wall and restore physiologic respiratory movement (Fig. 1d). Drains were placed over the pericardium and over the MegaDerm. They were removed on the 4th postoperative day and 6th postoperative day, respectively. There were no intraoperative or postoperative complications. The postoperative course was uneventful. The patient was discharged in good condition with relief from preoperative pain on the 8th postoperative day. The final pathology of the resected tumor confirmed metastatic HCC.

**Fig. 1:**
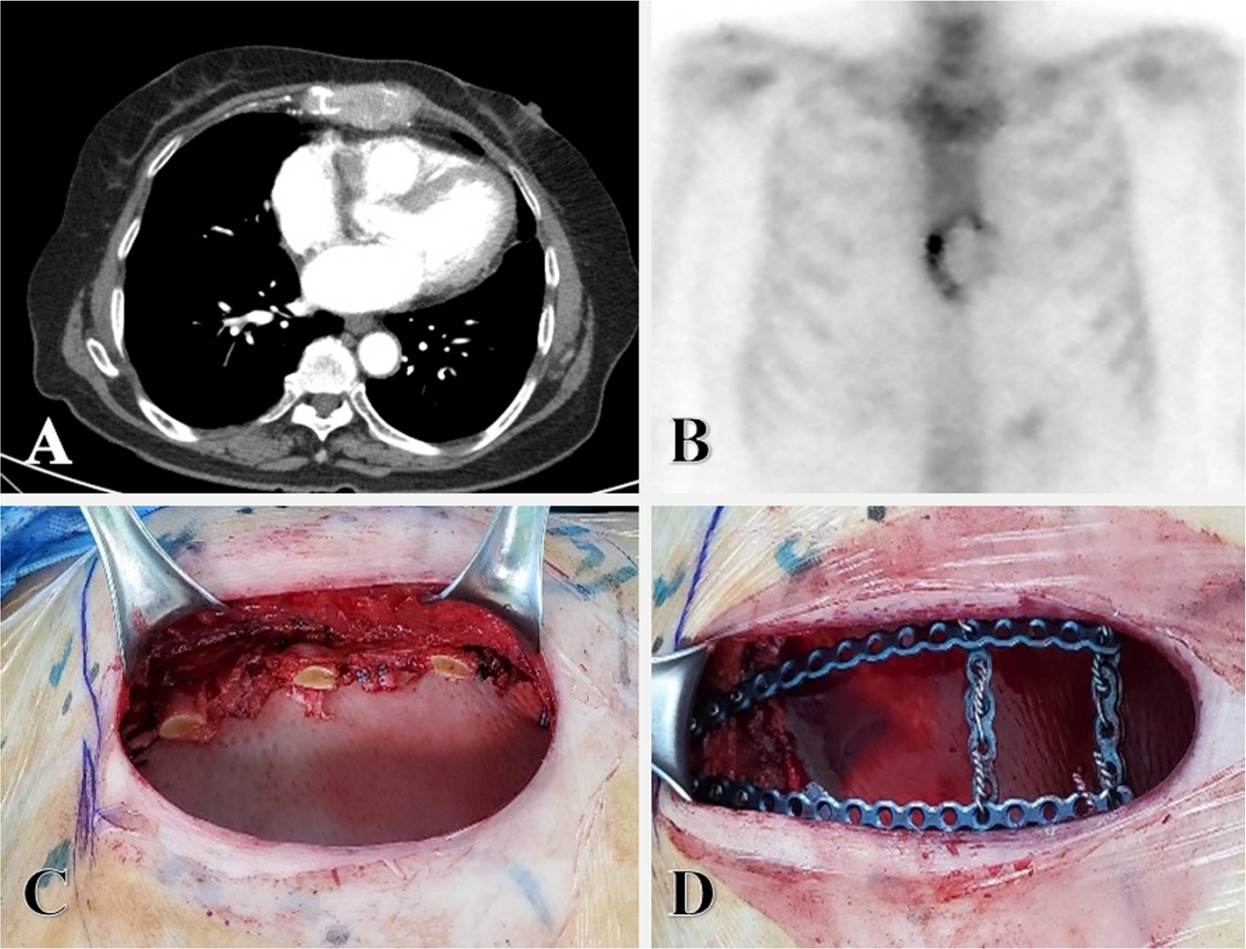
**a** Preoperative chest computed tomography and (**b**) whole body bone scintigraphy showing the tumor in the lower sternum. Intraoperative view of the anterior chest wall reconstruction with (**c**) acellular dermal matrix and (**d**) titanium plates

Unlike primary sternal tumors, there is no consensus on the treatment for metastatic sternal tumors because of their low incidence and limited data [2]. Some authors have suggested that surgical resection can provide survival benefits if patients with one or two isolated extrahepatic metastases simultaneously show good functional preservation of the liver and favorable performance conditions. Surgical resection can also successfully treat intrahepatic HCC [5].

Large defects after radical resection of the anterior chest wall need reconstruction to protect intrathoracic organs and restore physiologic chest wall movement. A variety of reconstruction techniques using various materials have been reported. However, definitive guidelines for the reconstruction of chest wall defects have not been reported [6]. Materials used in reconstruction include synthetic materials (polytetrafluoroethylene and polypropylene), biologic materials (acellular dermal matrix), metallic materials (titanium), and allograft/homograft. Each has its advantages and disadvantages. Polypropylene mesh is widely used due to its solidity, manageability, long-term solubility, low-frequency foreign body reaction, and low infection rates. However, it is used as a sandwich technique with methylmethacrylate because covering the defect with only polypropylene mesh is a somewhat weak cover for a significant defect and molded methylmethacrylate alone is hard to be fixed to adjacent bones. The first layer of polypropylene mesh is fixed directly to the base of the chest wall defect. Then molded methylmethacrylate is added to the defect as the second layer of prosthesis and third layer of polypropylene mesh is covered to fix the molded methylmethacrylate [1, 6]. Acellular dermal matrix consists of organic collagen-based matrix which stimulates regeneration by allowing for native tissue re-growth and revascularization. Unlike synthetic material, it can be placed directly over the lung and viscera without complications. However, the achieved stability does not result in a rigid reconstruction of the chest wall [6]. Surgical wound complications are crucial factors when selecting reconstruction materials. Some authors have reported no difference in the occurrence of surgical wound infections between the use of acellular dermal matrix and polypropylene for the skeletal chest wall reconstruction [7]. However, incidence of surgical wound complications including infections, wound dehiscence, skin necrosis, pneumothorax, pleural effusion, seroma, and hematoma are low when acellular dermal matrix is used [7]. Titanium has high corrosion resistance, low specific weight, and remarkable traction resistance. It is biologically inert and highly biocompatible [6]. Another sandwich reconstruction technique using titanium plates and biologic meshes, based on that the characteristics of biologic mesh consent its safe use with a second prosthetic material, has been reported [8]. This technique fixes titanium plates to the resected sternum and ribs between two layers of biologic meshes. The inner mesh is used to protect intrathoracic organs and the middle metallic plate is used to create an anatomic appearance and physiologic movement of the chest wall. The outer mesh is used to reconstruct the muscular plane. Biologic matrix is safely used with the titanium plate to create a precise shape and provide excellent reinforcement for defects due to its uniform tensile strength. It also creates an ideal substrate to avoid lung herniation and damage [8]. In our case, we performed radical resection of the sternum and costal cartilages without excising the soft tissues over the sternum. Reconstruction of significantly large anterior chest wall defects used an inner acellular dermal matrix, middle titanium plates, and the outer musculocutaneous tissue of the patient.

In summary, we encountered a rare case of metastatic HCC of the sternum that occurred one and a half years after hepatectomy. We radically resected the sternal tumor and reconstructed the anterior chest wall defect with acellular dermal matrix and titanium plates. The patient’s postoperative course was uneventful. Her pain subsided. Long-term surveillance is needed to determine the survival benefit of this surgery.

## Data Availability

Not applicable.

## Abbreviation

HCC: Hepatocellular carcinoma

## References

[1] Motono N, Shimada K, Kamata T, Uramoto H (2019). Sternal resection and reconstruction for metastasis due to breast cancer: the Marlex sandwich technique and implantation of a pedicled latissimus dorsi musculocutaneous flap. J Cardiothorac Surg.

[2] Dudek W, Schreiner W, Horch RE, Sirbu H (2018). Sternal resection and reconstruction for secondary malignancies. J Thorac Dis.

[3] Wu CY, Su CW, Lee FY (2011). Hepatocellular carcinoma with sternal metastasis. Clin Gastroenterol Hepatol.

[4] Kim SU, Kim DY, Park JY, Ahn SH, Nah HJ, Chon CY (2008). Hepatocellular carcinoma presenting with bone metastasis: clinical characteristics and prognostic factors. J Cancer Res Clin Oncol.

[5] Chan KM, Yu MC, Wu TJ, Lee CF, Chen TC, Lee WC (2009). Efficacy of surgical resection in management of isolated extrahepatic metastases of hepatocellular carcinoma. World J Gastroenterol.

[6] Sanna S, Brandolini J, Pardolesi A, Argnani D, Mengozzi M, Dell'Amore A (2017). Materials and techniques in chest wall reconstruction: a review. J Vis Surg.

[7] Giordano S, Garvey PB, Clemens MW, Baumann DP, Selber JC, Rice DC (2020). Synthetic mesh versus acellular dermal matrix for oncologic Chest Wall reconstruction: a comparative analysis. Ann Surg Oncol.

[8] Sandri A, Donati G, Blanc CD, Nigra VA, Gagliasso M, Barmasse R (2020). Anterior chest wall resection and sternal body wedge for primary chest wall tumour: reconstruction technique with biological meshes and titanium plates. J Thorac Dis.

